# Cultural Features Influencing Eating, Overweight, and Obesity in the Roma People of South Bohemia

**DOI:** 10.3390/nu10070838

**Published:** 2018-06-28

**Authors:** Věra Olišarová, Valérie Tóthová, Sylva Bártlová, František Dolák, Alena Kajanová, Dita Nováková, Radka Prokešová, Lenka Šedová

**Affiliations:** 1 Institute of Nursing, Midwifery and Emergency Care, Faculty of Health and Social Sciences, University of South Bohemia in Ceske Budejovice, J. Boreckeho 1167/27, 370 05 Ceske Budejovice, Czech Republic; tothova@zsf.jcu.cz (V.T.); sbartlova@zsf.jcu.cz (S.B.); fdolak@zsf.jcu.cz (F.D.); novakovad@zsf.jcu.cz (D.N.); lsedova@zsf.jcu.cz (L.Š.); 2 Institute of Social and Special-paedagogical Sciences, Faculty of Health and Social Sciences, University of South Bohemia in Ceske Budejovice, J. Boreckeho 1167/27, 370 05 Ceske Budejovice, Czech Republic; kajanova@zsf.jcu.cz; 3 Institute of Legal Branches, Management and Economics, Faculty of Health and Social Sciences, University of South Bohemia in Ceske Budejovice, J. Boreckeho 1167/27, 370 05 Ceske Budejovice, Czech Republic; rprokes@zsf.jcu.cz

**Keywords:** the Roma, nurse, overweight, obesity, health education, lifestyle factors, nutrition, cultural features

## Abstract

This article describes the important cultural specifics that impact on treatment of overweight and obesity for the Roma people of South Bohemia. Methods: Data on health and nutrition were collected using a semi-structured interview of 302 Roma adults (quantitative phase). A further 25 participants received in-depth interviews regarding their eating and lifestyle habits and perceptions about obesity and overweight (qualitative phase). Height and weight were measured with calibrated scales and stadiometer. Qualitative data were analyzed with the “grounded theory” method. Results: The participants reported a relatively high consumption of high-sugar drinks and foods compared to fruits and vegetables. Lifestyle factors increasing risk of overweight identified from the qualitative interview included unemployment, socially isolating housing, poor transport, poverty, inactivity, tobacco smoking, and for women weight gain after childbirth. Also identified was the need for better health literacy. Conclusions: Effective health education may help to address risk factors for overweight and obesity in Roma peoples. Other measures include improved socioeconomic status and housing security, and improved health literacy of the Roma people.

## 1. Introduction

Determinants of obesity include genetic predisposition, environmental factors, and the social-cultural environment [1,2,3]. 

Overweight and obesity date perhaps as far back as prehistoric times, i.e., as long as people have dealt with both an excess and a scarcity of food. However, over the course of societal development and with advances in scientific and medical knowledge, the social and cultural interpretations of diagnoses of overweight and obesity have changed as well. Although they are symbols of affluence in some societies, they also indisputably impact on health. According to available data, more than half of the population (52%) of the European Union are overweight or obese, and in the Czech Republic, 61.7% of the population have a body mass index (BMI) ≥ 25 kg/m^2^ [4]. Although the number of adult individuals with a BMI ≥ 25 kg/m^2^ seems to have levelled off, the proportion of people suffering from obesity is still rising [5]. 

The high rates of overweight are also evident in minority groups such as the Roma, who are one of the largest minorities of the Czech Republic [6,7]. Their number is estimated at 250–280,000 persons [8]. Studies designed to monitor the health status of the Roma show that, in spite of government efforts across Europe, they suffer from poorer access to health care and are often discriminated against [9]. Vazarova de Courten et al. [10] confirmed a potential link between the high prevalence of obesity with unhealthy lifestyles and improper eating habits. One study that looked at the incidence of overweight and obesity in Roma women in Slovakia found similar results. It was found that Roma suffer from extreme degrees of obesity much more often than the rest of the population. In these cases, the genetic predispositions of susceptible individuals are also supported by unhealthy lifestyles [11]. 

Together with external determinants, persistent cultural characteristics of the Roma can notably influence the efficiency of the public and other health interventions [12,13]. The Roma are a distinctive group with distinctive cultural features, language, and a singular cultural identity, setting them apart from the majority of the population [14]. Over the course of the centuries, they spread all over the world thanks to their nomadic lifestyle. Due to encounters with the dominant culture, there have been changes in their traditional way of life, but some particularities persist [15,16]. These particularities are evident primarily in the way of life, eating habits, experiences and perceptions of time, socio-economic status, and poorer access to services or health [14]. Preventive programs, in order to be effective, need to consider diverse cultural determinants of health and, above all, the individual needs of the target group [17]. At the same time, the level of nutritional acculturation or the level of adoption of the nutritional habits of the majority population by immigrants or other minority groups should be taken into account [18]. The preventive programs implemented at present with focus on overweight and obesity is developed for the dominant population in the Czech Republic. Their effectiveness for the Roma appears to be very low. 

This article aims at to explore cultural features of the Roma that could be associated with their high prevalence of overweight and obesity and the inefficiency of currently implemented interventions. First, the anthropometrics status, nutritional intake, and eating patterns of a group of Roma participants were investigated. This was followed by exploratory interviews investigating lifestyle, intake, and sociodemographic features of a subsample of respondents. 

## 2. Materials and Methods

### 2.1. Quantitative Phase 

Participants were sourced from the Roma population in the South Bohemian Region between 1 June 2014 and 31 March 2015. General nurses from the Centre for Prevention of Life Style Diseases in České Budějovice recruited participants at community events and in the respondents’ natural environment with the assistance of contact persons. Because census data is not available for the Roma minority in the Czech Republic, respondents were selected using the snowball method aiming to ensure an equal representation of men and women (as in the general population in the Czech Republic). Further, the respondents from the Roma minority were selected based on their subjectively perceived Roma identity. 

Face-to-face semi-structured interviews were conducted using a prepared non-standardized questionnaire. The communication with the respondents was performed in Czech. The respondents were given primarily close-ended questions with employment of scaled answers. Further, the following anthropometric values were obtained: weight (determined with electronic flat scale SECA 813); height (determined with a SECA 213 folding height meter); waist and hip circumference (measured with tape measure); and body fat content (measured with OMRON BF306 Body Composition Monitor). Body mass index (BMI; kg/m^2^) was subsequently calculated. 

BMI reference values for obesity were applied according to those of the World Health Organization (WHO), i.e., BMI 18.5–24.9 kg/m^2^ normal, BMI 25–29.9 kg/m^2^ overweight, and ≥30 kg/m^2^ obese [19]. Reference values suggesting risk metabolic complications were applied for waist circumference: men, ≥94 cm, suggesting increased risk, and ≥102 cm, suggesting very high risk; women, ≥80 cm, suggesting increased risk, and ≥88 cm, suggesting very high risk [19]. 

Three hundred and two Roma individuals over 18 years of age participated; males and females were equally represented. Most were aged 18–29 years (132, i.e., 43.8%); 56 (18.6%) were aged 40–49 years, 49 (16.3%) were aged 30–39 years, 39 (13%) were aged 50–59 years, 20 (6.6%) were aged 60–69 years (20, i.e., 6.6%), and only 5 (1.7%) were aged ≥70 years. There were more respondents who had three or more children (*n* = 134, 44.3%) than those who had no children (*n* = 77, 25.6%). One hundred and forty-nine (50.1%) persons were unemployed and only 51 (17.2%) were practicing a profession. The others received a disability or old age pension or were on maternity leave. 

### 2.2. Analyses

Data analysis was carried out with using the SASD (Statistical Analysis of 129 Social Data) program and SPSS program (in version 13.1). Statistical associations were tested with the Pearson Chi-square test—*X*^2^) and significance levels of α < 0.05, α < 0.01 and α < 0.001. In case of insufficient observations, Yates correction was applied. 

### 2.3. Qualitative Phase 

The qualitative research was aimed at acquiring more in-depth information regarding attitudes the Roma minority to overweight and obesity. It was also aimed at mapping the nutritional habits and describing the risk factors with respect to culturally conditioned behavior patterns occurring in the natural environment (Roma households and communities). The qualitative research set consisted of 25 Roma respondents over 18 years of age (8 males and 17 females). The male sample included six overweight individuals (BMI ≥ 25 kg/m^2^) and two obese ones (BMI ≥ 30 kg/m^2^); ages ranged from 19 to 64 years. The female sample included eight overweight individuals and nine obese individuals; ages ranged from 29 to 64 years. 

Participants received a semi-structured interview; this comprised a circle of 35 basic questions with 90 sub questions in advance, and their order could be changed to achieve the highest possible interview efficiency. The interview scheme prepared in this manner included 35 basic questions divided into four circles: eating habits, physical and leisure-time activities (lifestyle), and self-perception and perceptions of overweight and obesity. The interviews took place from April 2014 to April 2015 in the community, primarily in individual’s homes. They were conducted in Czech and complemented by anthropometric measurement (height, weight, calculation of BMI, waistline and body fat measurements as above). The interviews made particular use of open-ended questions. The interviews were subsequently digitalized and transcribed verbatim. The MAXQDA 11 (Software for Qualitative and Mixed Methods Research) program was chosen for the text analysis. One thousand and eight-two segments in total were marked in the transcribed interviews, subsequently exported, and further processed. To analyze the data, we used “grounded theory” including open, axial, and selective coding. Finally, we identified the central category—lifestyle. From among other categories, we identified eating habits, motivation, attitude and body image. To ensure anonymity, the respondents were given randomly selected names when processing the data. 

## 3. Results 

### 3.1. Quantitative Phase 

The analysis of the data related to the assessment of the basic health indicators suggests that in the research set including 302 Roma from the South Bohemian Region, 61.8% persons had BMI ≥ 25 kg/m^2^. Overweight (BMI 25.0–29.9 kg/m^2^) was found in 29.7% of participants and obesity (BMI ≥ 30 kg/m^2^) in 32.1% of respondents. It was proved that there were statistically significant differences between males and females in terms of BMI index (*p* < 0.01; *X*^2^ = 11.17; df = 2). The males had lower BMI index significantly more often, while the females were overweight (BMI 25.0–29.9 kg/m^2^) and obese (BMI ≥ 30 kg/m^2^) more often. 

From among further indicators of overweight and obesity, the waistline values were observed. Increased values were recorded. One-third of the examined Roma (*n* = 152) had a waistline exceeding 102 cm. It should be stated here that males’ waistline values over 102 cm are related with noticeable metabolic complications. Waistline values related to noticeable metabolic complications (over 88 cm for females) were also found in Roma women (63.3%, *n* = 150). Also, the measured values of body fat correspond to the above stated indicators. The difference between genders was minimal, but the higher values of body fat of the Roma (found in 67.55% of respondents) in the South Bohemian Region were measured. 

From the perspective of prevention of overweight and obesity, the information related to consumption, selection, preparation and intake frequency of foodstuffs is important. We show the preference of selected foodstuff types in the Roma minority (Table 1) below. 

As shown on Table 1, chicken was the most frequently consumed meat, followed by pork and then beef. More than half of the respondents (56.2% persons) did not eat fish. “Side dishes” of potatoes, pasta, rice and dumplings were most commonly eaten 1–2 times a week, followed by 3–4 times a week. Fruits and vegetables were consumed at a lower frequency: most often either not at all or only 1–2 times a week. With respect to sweet baked goods, those most frequently consumed were cakes and doughnuts, but also pies, and cakes. Sweets included different types of wafers, ice cream, biscuits, chocolate and candies. 

In relation to the energy balance, we also consider significant the findings related to consumption of sweet drinks (like cola, juices, water with squash, energy drinks, etc.). In this case, analysis showed that the members of the Roma minority drink soft drinks in higher frequency. 

### 3.2. Qualitative Phase

The qualitative phase was implemented to explore motivational factors and culturally conditioned behavior patterns with respect to prevention and treatment of overweight and obesity. The qualitative data were analyzed by the MAXQDA 11 program. We worked with transcribed interviews. In this study, 1082 interview segments were marked in total, exported and analyzed. The analysis was made using “grounded theory” use of open, axial and selective coding [20]. The terms found were grouped by their potential belonging to categories (open coding). However, at that point, the categories could not be considered final, as their belonging to any given phenomenon was only apparent. The conceptual range of the categories gave base to their contents. We show an example in the table (Table 2).

The open coding allowed us to divide the acquired data and to determine some categories and their characteristics. Axial coding allowed us to put the data together in a new way by creating links between the categories and their subcategories. As an example, we show the embedding of the “body image” category into the paradigmatic model (Table 3). 

Visual inspection of the paradigmatic model showed that the circular dimension best explains phenomena, as consequences sometimes may mingle with causal conditions. 

Based on the acquired overview of the relations among individual categories and subcategories, the codes found were subjected to a deeper analysis. The analysis helped to identify the central category of “lifestyle”, to which all other categories are related. Lifestyle constitutes the phenomenon that is closely related to eating habits, motivation, and attitude to overweight and obesity, as well as to body image. It is connected not only with the way of spending leisure time, risk behaviors, and the way of spending weekdays, but it also reflects the cultural perspective. Its characteristics influence individual areas of eating habits (e.g., choice of foodstuffs, regularity of eating, etc.). The factors resulting from it are reflected in the motivation to reduce weight, and in the individual’s attitude to overweight and obesity. In this point, they are closely related to self-perception and to body image, as well as to life satisfaction. 

To achieve integration of the findings, the story skeleton was interpreted, and, upon this basis, a scheme of the relations found was created (Supplementary Figure S1). 

#### 3.2.1. Story Skeleton 

Remedial risk factors for overweight and obesity are related to lifestyle in the Roma population. The vast majority of the respondents were unemployed, women who were at home with children, and some men receiving the disability or old-age pension. This factor undoubtedly plays a role in the distribution of activities during the day. Their weekdays are related primarily to cleaning, eating, caring for children, and community life. Most of those Roma live in socially excluded localities, in residence halls far from the town center. They have poor transport access. Their life is noted for risk behaviors like heavy smoking and inactivity. Consumption of alcohol is rather occasional, although there are exceptions too. Poor access to transport influences their eating habits, primarily due to the accessibility of groceries. 

The above-stated distribution of daily activities, normal times of getting up, and factors of social life in broader family living in the given community influence eating habits such as frequency and regularity of eating. The Roma report eating at home and in the homes of relatives whom they visit. Some of them have even multiple main dishes. Nevertheless, they have problems with describing their meals. It is difficult for them to tell how many times a day they eat. Great differences can be seen when they describe their meals of the previous day. It is effective to make use of the opportunity to describe the course of the day and to encourage them to recount what they did from the time they woke up in the morning. Then they are able to approximately describe the foodstuffs and liquids they took in. As for foodstuffs and liquids, they use illustrative comparison and examples, which they can grasp better. Their composition of foodstuffs does not essentially differ. They show a tendency to give more healthy foodstuffs (fruits and vegetables) to their children. The socioeconomic perspective is often mentioned here. We can see the financial issue. They prefer to buy something for the children, and if some money is left, they buy something for themselves too. They agree in the traditional Roma meals. However, they report their intake in a frequency of several times a month. 

They are aware of the causal relation between eating and increasing weight. The women often restrict food intake to decrease their weight. However, they do not show long-lasting eating changes or dietary measures related to other diseases they suffer from. They observe regime recommendations only until the acute symptoms subside; as for medicines, they take them only until they finish the package. They do not go to the physician to ask for more. Women do not include significant amounts of exercise in their lifestyle; they exercise primarily if the physician has suggested so because of backache. On the contrary, men make use of short-term exercise in fitness centers, trying to improve their physical condition. Some of them are aware of the positive effect of exercise and sports. The increased weight of women is primarily related to childbirths after which they did not succeed in reducing their weight, although many desire to lose weight. When assessing their own figure, particularly the women tend to be undervalue their weight; they perceive themselves slimmer than they actually are. On the contrary, men are usually more objective. 

The self-perception is related to life satisfaction that is reflected also in the lifestyle and affects motivation. The Roma are rather satisfied with their family lives; they perceive themselves as cheerful and quick-tempered persons. However, their life satisfaction is affected by housing, primarily by the life in communities and socially excluded localities. Many of them are not satisfied with such a lifestyle. They do not perceive the difference between overweight and obesity, they relate them rather to a disease or overeating. Most of them do not take any attitude do such people; they do not criticize them, but some feel sorry for them. Some motivation to reduce the weight consists primarily in weight reduction recommended by physicians, and women also consider the aesthetic side, if they cannot dress what they like or if they feel they compare poorly next to their partner. 

#### 3.2.2. Diagram 1 Visualization of Relations

The visualized relations helped us to create the following categories: lifestyle, eating habits, motivation, attitude, and body image. Lifestyle was identified as the central category (Table 4). Based on the connections found, we came to the conclusion that the prevention and treatment of Roma overweight and obesity are influenced by culturally conditioned influenceable risk factors resulting from their lifestyle. To eliminate them, adequate motivation and efficient education should be used. Indispensable measures include motivation to long-lasting changes, to increasing health literacy of the Roma and regular monitoring of basic health indicators. However, the impact of the other risk factors cannot be omitted in the given context. The risk factors include primarily lack of exercise, risk behaviors (smoking), socioeconomic factors related to financial security, and housing and life in socially excluded localities. 

## 4. Discussion

The prevalence of obesity in Europe is regularly confirmed in a range of 10–40% of the population. In addition to a genetic predisposition, the main causes include lifestyle and underestimation of the seriousness of the complications related to this disease [21]. Further determinants are: influence of the environment, improved economic situation of individuals or ethnic differentiation. The economic situation is closely related to education and income level of the population. Thus, it can be stated that the obesity in highly developed countries, including the Czech Republic, is more frequent in inhabitants with lower education and lower income, as well as in rural populations. On the contrary, the obesity in developing countries is rather related with higher social status and social prestige [5,22]. With regards to ethnicity, it is assumed that ethnicity is often a more significant predictor for overweight and obesity in children than social class or other socio-economic variables [23,24,25,26]. The results of the present study clearly suggest that, according to the above stated indicators, the Roma are at increased risk of obesity. 

Normal waistline values were found in 21.4% of Roma females in the examination. In the present study the majority of the Roma participants had increased waist measurements and high BMI. Reported lifestyle factors such as unemployment and late rising (often at 10–11 a.m.) likely also contribute to obesity risk. Similarly, the qualitative findings found Roma to be underemployed with a lifestyle of sedentary activities (sitting at the computer, watching TV, contact with friends and relatives, or sleeping). However, some were aware of the positive effect of exercise and reported that when they exercised, they had less backache or pains of legs and arms. 

Risk behaviors—primarily smoking—are determined by lifestyle to some degree. The respondents of the qualitative phase included more than one half of smokers; the smokers could be divided into two groups. The first group included persons who smoked less than 10 cigarettes a day; the second (comparably large) group would include person who smoke more than a pack of cigarettes a day. A distinct representation of smokers among the Roma is reported also by Urban and Kajanová [27]. They examined a sample of 164 Czech Roma and found 63.5% of males were smokers, and 58% of females. They add that the respondents started smoking at 14 years of age on average. The results related to consumption of alcohol were a little more positive. Occasional or no consumption prevailed among the respondents.

The area of nutrition of the Roma minority is described well in the following quotation [28]: “The Roma may be as poor as a church mouse, but must have enough food, even if there may be nothing left for tomorrow.” These words suggest the usual eating habits. Eating is not a regular ritual, but it is closely related to the status of the family [12,29]. At present, the Roma families cook a lot, particularly after the payment of wages of social or other benefits. The parents often try to give their children everything they were deprived of. Children’s food is usually not distinguished from the adults’ food; meat, smoked meat products, and sweets are mostly consumed. Fruits and vegetables are not frequently consumed [30]. A very low intake of fruits and vegetables is confirmed also by Stávková and Derflerová Brázdová [31]. They also reflect on the issue of nutrition literacy, as their study showed that only 15.7% of the Roma respondents (*n* = 102) knew the recommendations with respect to minimum fruit and vegetable intake. Consumption of fruits and vegetables was also low, and most were cooked: “Well, yes, I eat them. We always have them. Raw, no. Not even salad, nothing. Only in meals. No, not every day, about twice a week” (Jolana). The most frequently consumed vegetables included tomatoes, peppers, cucumbers and carrots. In contrast, the traditional Roma meals constitute an integral part of the Roma diet. Oláhová [28] includes goja and flour pancakes among them. Very thick soups are reported by Davidová [30]. Our respondents reported, additionally to goja, also halusky, filled cabbage, pork with dumplings and fasulja, a traditional thick soup. They also reported eating such meals only several times a month, i.e., they did not perceive their consumption as too frequent. One of the female respondents characterized the Roma cuisine as follows: “The Gypsies have the marikl’a, halušky, fasulja (that is bean soup). We love meat. You can see meat, schnitzel, pork on Gypsy tables. Just meat, fatback, dumplings, pork, yeah. Meals of that kind. Pishot is a Gypsy meal too. Sweet triangles with cream cheese and marmalade. And the Gypsies have four to five meals, just a lot of food. And the Gypsies are used to put food on the table, if they have it. You know, we like eating. But attention, the Gypsies love oil, fat.” (Irena). The above stated characteristics of traditional Roma cuisine may explain, to some degree, the preference for some side dishes (e.g., potatoes, dumplings, etc.). The analysis of the qualitative data also suggested the importance of attitude to overweight and obesity. This is primarily a result of the respondents’ own experiences and health conditions, and is influenced by sociocultural background. A possible relationship between the social pressure directed at the preferred physical appearance across different cultures, and between the determinants like education, degree of acculturation, or BMI is confirmed also by Cachelin et al. [32]. Although our respondents did not perceive the difference between overweight and obesity, many of them had their own experience of the consequences of such conditions. They most frequently reported leg pain, breathlessness (primarily when climbing stairs and running to catch the bus), and backache. Some women mentioned also the impact on the individual’s appearance, which is, however, not necessarily sufficient motivation for change: “Well, great. Primarily appearance. Health impact not much, well, the legs, nothing. But only when I looked at the mirror, nothing suited me. I took it off.” (Irma), “Well, you cannot dress everything; although now I have lost some weight.” (Irena). 

The Roma may be at increased risk of eating disorders as well as higher weight through factors such as less regular eating and a culture of moderate overconsumption. Culturally-based behavior patterns appear to be very important determinants of eating habits. Also evident is the impact of perception of overweight and obesity, body image, lifestyle, and in living in socially excluded localities. On the other hand, the decreased regard for health benefits of weight loss may be protective. Further studies are needed to examine disordered eating behaviors such as binge eating in Roma and whether increased exposure to the ‘mainstream’ attitudes to weight and shape may result in an increase in eating disorders.

## 5. Conclusions

Remedial risk factors for overweight and obesity in the Roma respondents we approached included primarily inactivity, lifestyle, and inappropriate structure of food together with eating habits and, to some degree, socioeconomic factors such as living in socially excluded localities. It has also been found that the Roma do not differentiate between overweight and obesity. They call both conditions “fat” and they often related increased weight primarily to a disease or to overeating. It is unknown to what degree these attitudes may however protect them from eating disorders common in the other European populations. 

## Figures and Tables

**Table 1:** Preference of selected foodstuffs in the Roma minority per week (*n* = 302).

	Intake Frequency, *n* (%)						
Selected Foodstuff	Not at All	1–2 Times a Week	3–4 Times a Week	5–6 Times a Week	Every Day	Several Times a Day	Did Not Answer
Pork	28 (10.3%)	112 (41.3%)	96 (35.5%)	21 (7.7%)	10 (3.7%)	4 (1.5%)	31 (10.3%)
Beef	82 (32.0%)	148 (57.8%)	19 (7.4%)	5 (2.0%)	1 (0.4%)	1 (0.4%)	46 (15.23%)
Fish	158 (56.2%)	106 (37.7%)	10 (3.6%)	2 (0.7%)	3 (1.1%)	2 (0.7%)	21 (6.95%)
Chicken	16 (5.8%)	125 (45.3%)	98 (35.5%)	25 (9.1%)	7 (2.5%)	5 (1.8%)	26 (8.61%)
Potatoes	14 (5.0%)	157 (56.4%)	79 (28.3%)	14 (5.0%)	11 (3.9%)	4 (1.4%)	23 (7.62%)
Pasta	17 (6.3%)	191 (70.4%)	40 (14.8%)	15 (5.5%)	7 (2.6%)	1 (0.4%)	31 (10.26)
Rice	24 (8.9%)	186 (68.9%)	42 (15.6%)	12 (4.4%)	4 (1.5%)	2 (0.7%)	32 (10.6%)
Dumplings	22 (8.1%)	181 (67.1%)	49 (18.1%)	11 (4.1%)	6 (2.2%)	1 (0.4%)	32 (10.6%)
Sweet baked goods	53 (18.5%)	126 (44.2%)	57 (19.9%)	8 (2.8%)	39 (13.6%)	3 (1.0%)	16 (5.3%)
Sweets	46 (15.9%)	125 (43.3%)	46 (15.9%)	15 (5.2%)	50 (17.3%)	7 (2.4%)	13 (4.3%)
Sweet drinks	52 (18.1%)	80 (27.8%)	53 (18.4%)	19 (6.6%)	75 (26.0%)	9 (3.1%)	14 (4.64%)
Fruits	38 (13.5%)	122 (43.4%)	61 (21.7%)	9 (3.2%)	37 (13.2%)	14 (5.0%)	21 (7.95%)
Vegetables	30 (10.8%)	96 (34.7%)	64 (23.1%)	23 (8.3%)	53 (19.1%)	11 (4.0%)	25 (8.27%)

**Table 2:** Open coding.

Category	Subcategory	Characteristic
Food intake	Motivation for foodstuff selection	
	Preferred foodstuff preparation	
	Place of alimentation	
	Number of meals a day	
	Frequency of alimentation	
	Regularity	Breakfast
		Morning snack
		Lunch
		Afternoon snack
		Dinner

**Table 3:** Application of paradigmatic model.

Causal Conditions	Phenomenon	Context	Intervening Conditions	Action Strategy	Consequences
Weight Health condition Self-perception Subjective assessment of own figure Life satisfaction	Body image	Feeling Body proportions Health complications Weight deviations Nature Family Housing	Unemployment Housing conditions Culturally conditioned behavior patterns Insufficient health literacy Number of children	Exercise Dietary measures	Temporary weight reduction Improvement of health complications Better self-feeling

**Table 4:** Resulting categories.

Category	Subcategory
Life style	Weekday Leisure time activities Prevention Risky behavior
Eating habits	Food components Traditional cuisine Food intake
Motivation	Reduction efforts Consequences
Attitude	Perception of overweight and obesity Preference of figures
Body image	Weight Life satisfaction

## Supplementary Materials

The following are available online at http://www.mdpi.com/2072-6643/10/7/838/s1, Figure S1: Visualization of relations.
