# Supplementary figures and images for: Cultural Features Influencing Eating, Overweight, and Obesity in the Roma People of South Bohemia
Source: Nutrients. 2018 Jun 28;10(7):838. doi: 10.3390/nu10070838 (PMC6073144; doi:10.3390/nu10070838)

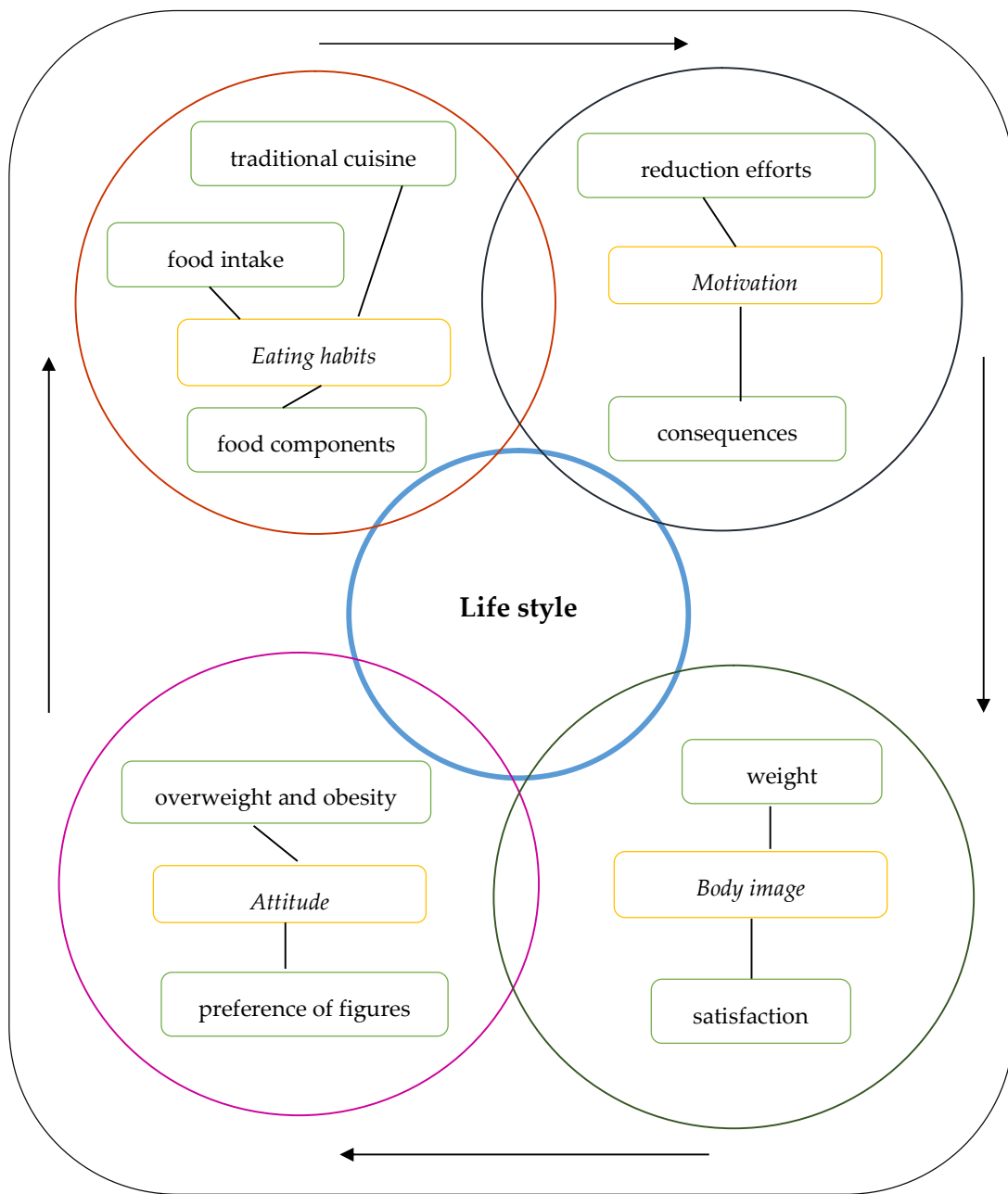

Figure S1. Visualization of relations.

Supplement: Supplementary file 1 [file nutrients-10-00838-s001.pdf]
